# Beyond the *Prdm9* model: independent evolution of hybrid male sterility in house mice

**DOI:** 10.1038/s41437-026-00834-9

**Published:** 2026-03-12

**Authors:** Pavla Klusáčková, Agata Woźniewska, Petra Dufková, Beth L. Dumont, Jan M. Wójcik, Jaroslav Piálek

**Affiliations:** 1 Research Facility Studenec, Institute of Vertebrate Biology Brno, Czech Academy of Sciences, Brno, Czech Republic; 2 Department of Botany and Zoology, Faculty of Science, Masaryk University, Brno, Czech Republic; 3 Mammal Research Institute, Polish Academy of Sciences, Białowieża, Poland; 4 The Jackson Laboratory, Bar Harbor, ME USA

**Keywords:** Evolutionary genetics, Evolutionary biology, Speciation

## Abstract

Hybrid sterility is a critical postzygotic barrier that limits gene flow during speciation, yet the genetic architecture underlying evolution of such barriers in the early stages of speciation remains poorly characterized. In house mice, F1 male sterility observed in crosses between *Mus musculus musculus* and *M. m. domesticus* has been attributed to incompatibilities between heterozygous autosomal *Prdm9*, which controls primarily the position of recombination hotspots, and copy number variation in X-linked *Mir465* miRNA genes. This molecular mechanism, identified in laboratory crosses, provided the first genetic evidence of a Dobzhansky–Muller incompatibility causing F1 hybrid sterility in vertebrates and has been considered a general model across strains and laboratories. Here, we use mice from natural populations and find that F1 hybrid sterility is polymorphic and asymmetric, with fertility phenotypes modulated by the direction of the cross. Although sterile males carried incompatible *Prdm9* alleles, quantitative trait loci (QTL) mapping in backcross progeny revealed no significant associations with chromosome 17, where *Prdm9* resides. Instead, sterility consistently mapped to X-linked loci, and the genomic position of sterility-associated QTL shifted between reciprocal backcrosses. These findings uncover a previously unrecognized mode of hybrid sterility in which X-linked incompatibilities act independently of *Prdm9*, a mechanism we term *Prdm9*-independent X-linked sterility (PIXLS). Our results extend the established *Prdm9*/*Mir465* model by demonstrating that hybrid sterility in house mice can arise through alternative genetic routes, highlighting the evolutionary diversity of reproductive barriers in their natural hybrid zone.

## Introduction

For speciation to occur, reproductive barriers must limit or prevent gene flow between diverging populations. Although considerable effort has been devoted to identify ‘speciation genes’ across diverse taxa, most such genes have been discovered in *Drosophila* (reviewed in Coyne and Orr 2004). However, because hybridizing fly species are typically highly diverged, it is difficult to determine whether these genes are true causal agents of speciation or whether they arose as a consequence of completed speciation (Mallet 2006). The house mouse (*Mus musculus*) provides a complementary system in which speciation can be studied at an earlier stage. Two subspecies, *M. m. domesticus* and *M. m. musculus*, diverged only around 200,000 years ago (Fujiwara et al. 2022) and, following colonisation from Western Asia, they came into secondary contact in central Europe. Today, they interbreed along a long but narrow hybrid zone, where incompatible genetic combinations are exposed and removed by selection (Ďureje et al. 2012; Janoušek et al. 2012; Macholán et al. 2007). Two studies from independent transects across the hybrid zone revealed that males from its central regions show reduced sperm counts and motility compared with individuals from the parental subspecies (Albrechtová et al. 2012; Turner et al. 2012). These findings suggest that genetic incompatibilities disrupting spermatogenesis can act as particularly effective barriers to gene flow, thereby maintaining divergence between the mouse genomes.

Experimental crosses between laboratory mouse stocks (mostly representing the *domesticus* genome) and wild *musculus* mice provided a direct demonstration of such barriers, producing sterile hybrid males (Forejt and Iványi 1974). The first ‘speciation gene’ controlling this hybrid sterility has been identified as PR/SET domain 9 (*Prdm9*) (Mihola et al. 2009). The identification of *Prdm9* has enabled researchers to determine which allelic combinations lead to reduced fertility in intersubspecific hybrids. The gene primarily determines the position of recombination hotspots during meiosis through a zinc-finger (ZnF) minisatellite array, whose product binds to specific chromosome sites to initiate meiosis (Baudat et al. 2010; Myers et al. 2010; Parvanov et al. 2010). When incompatible alleles are present in F1 hybrids, the asymmetrical placement of methylation marks on histones can hinder the repair of double-strand breaks, resulting in increased chromosome asynapsis and spermatogenic arrest (Davies et al. 2016; Forejt et al. 2021). Two incompatible ZnF alleles were identified in laboratory crosses between a classical C57BL/6 mouse stock (B6, with a predominantly *domesticus* background) carrying the dom2 allele and a wild-derived PWD strain from the *M. m. musculus* subspecies possessing the mus1 allele (Mihola et al. 2009; Parvanov et al. 2010). This allelic combination, together with the X-linked *Hstx2* locus and *musculus*/*domesticus* autosomal heterozygosity are necessary components of the PWD^Prdm9mus1^/B6^Prdm9dom2^ F1 sterility model. In natural populations, sterility has also been observed in hybrids with the dom3 allele (found in wild-derived *domesticus* strains) and the mus1, mus2, and mus5 alleles from wild-derived *musculus* strains (Mukaj et al. 2020).

*Prdm9* does not act independently. In the PWD/B6 model, its epistatic interaction with the hybrid sterility X-linked (*Hstx2*) locus from the *musculus* lineage is essential. This locus has recently been identified as microRNA 465 (*mir465*) gene, which influences both meiotic chromosome synapsis and the rate of recombination (Jansa et al. 2025). Experimental crosses with different wild-derived strains have validated this F1 hybrid sterility model, supporting its broad relevance to wild mouse populations (Forejt et al. 2021; Good et al. 2008a; White et al. 2012). However, while both loci are necessary for sterility, they are not sufficient. *Musculus*/*domesticus* heterozygosity on short chromosomes is required to trigger sterility, which is further modulated by a locus in the centromeric region of the X chromosome. In backcross and F2 populations, homozygous stretches exceeding 27 Mb on short chromosomes are sufficient to restore synapsis and correlate with improved male fertility (Fotopulosova et al. 2024; Gregorová et al. 2018). This may explain the reduced impact of *Prdm9* interallelic incompatibility observed in experimental backcrosses and intercrosses (J. Forejt, unpublished data).

The genetic model underlying negative interaction between diverged genes aligns with the Dobzhansky–Muller model of speciation, which posits that the accumulation of incompatible alleles at two or more loci in diverging populations drives reproductive isolation (Dobzhansky 1941; Muller 1942). Studies in *Drosophila* have demonstrated that ‘speciation genes’ evolve rapidly under the influence of natural selection (Coyne and Orr 2004). In both humans and mice, the *Prdm9* gene displays high variability in natural populations and clear signatures of rapid evolution and positive selection (Ponting 2011). During the process of fixation, incompatible alleles are initially polymorphic, with positive selection progressively reducing allelic variability from local to population-wide scales. Thus, while global genetic variation reflects the degree of divergence, local sampling is expected to reveal reduced haplotype diversity. Comparable population data for the recently identified *Mir465* are not yet available, but preliminary evidence from wild-derived strains suggests that subspecies-specific structural variation may drive the evolution of this miRNA (Jansa et al. 2025). To assess how the allelic turnover at two loci is shaped by positive selection, population genetic data are essential. However, such data linked to fertility estimates remain lacking.

Despite significant progress in understanding mouse hybrid sterility, several key questions remain unanswered. To date, most research has relied on crosses between PWD and B6 inbred strains, with limited exploration of natural variation. Only a small subset of *Prdm9* alleles has been tested for their presumptive effects on fertility in intersubspecific hybrids (AbuAlia et al. 2024; Mukaj et al. 2020). Moreover, in crosses involving wild-derived strains, direct effects of novel allelic combinations in sterile F1 hybrids were not mapped for genomic correlates. Intriguingly, data from wild mice indicate that the sterility-inducing *Prdm9*^mus1/dom3^ allele combination can sometimes produce fertile offspring (Mukaj et al. 2020), underscoring the complexity of these genetic interactions and suggesting that the evolution of sterility in house mice may be more intricate than previously appreciated.

In this study, we provide evidence for an alternative genetic basis of sterility in wild mice by integrating population genetic data with fertility assays. We examined two local populations representing the European house mouse subspecies and found high allelic diversity at *Prdm9* in both sites, mirrored by polymorphism in fertility among F1 hybrids. Quantitative trait locus (QTL) mapping in backcross males revealed that sterility is not directly attributable to *Prdm9*. Instead, it is associated with X-linked loci, whose effects depend on the direction of the backcross. These findings provide new insights into the genetic architecture of sterility and highlight the importance of considering cross-type directionality in hybrid incompatibility studies.

## Results

### Microgeographic variation of *Prdm9*

**Fig. 1:**
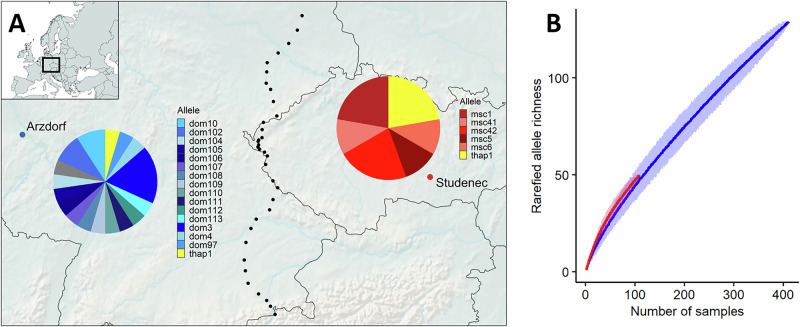
Allelic diversity in house mice. **A** *Prdm9* polymorphism in Arzdorf (Germany) and Studenec (Czechia). The pie charts indicate the frequencies of alleles named in the legend and detailed in Dataset S1. Black dots indicate the location of the house mouse hybrid zone, which is ~10 km wide (Macholán et al. 2007; Macholán et al. 2024). The inset map, showing the outline of Europe and indicating the position of study area, was created with MapChart (https://www.mapchart.net/). The main map was generated using the Free and Open Source QGIS (https://www.qgis.org/). **B** Rarefaction curves with bootstrap confidence intervals (shaded areas) for *Prdm9* allelic diversity in *M. m. domesticus* (blue) and *M. m. musculus* (red).

*Prdm9* allelic diversity was analysed in two localities, Arzdorf and Studenec, located 380 km and 250 km from the house mouse hybrid zone, respectively (Fig. 1 A). Given that the dispersal of house mice is approximately 1 km/year (Macholán et al. 2007), introgressive hybridization is unlikely to have influenced the genetic background of mice in these populations. Although human-mediated long-distance transport cannot be excluded, analysis of 5000 SNPs (see Materials and Methods) confirmed their assignment to native subspecific genomes. The *Prdm9* gene exhibited polymorphism in both localities. Sequencing of 21 mice from Arzdorf (*M. m. domesticus*) identified 17 alleles, while 9 mice from Studenec (*M. m. musculus*) revealed 6 alleles, resulting in local allelic diversities of 0.81 and 0.67, respectively (Dataset S1). Only one allele was shared between the two localities, and it was restricted to a 40-Mb inversion on chromosome 17, known as the *t*-haplotype, which predates the divergence of *Mus musculus* subspecies (Morita et al. 1992). Both populations harboured sterility-inducing *Prdm9* alleles, dom3 and mus1, at frequencies of 0.14 and 0.22, respectively. All sequences have been deposited in GenBank (accession numbers PV833228—PV833253).

To assess *Prdm9* diversity across *Mus* *musculus* subspecies, we compiled published data on *Prdm9* variability (Dataset S2). Among 403 M. m. domesticus samples, 124 alleles were identified, including 13 novel alleles detected in Arzdorf. Similarly, among 100 *M. m. musculus* samples, 42 alleles were identified, with two novel alleles discovered in Studenec. Although allelic diversity estimates calculated at the subspecies level yielded lower values (0.31 for *M. m. domesticus* and 0.42 for *M. m. musculus*), direct comparisons of local and subspecies-level diversity are potentially confounded by unequal sampling effort and heterogeneous study designs. To assess how completely current sampling captures natural *Prdm9* allelic diversity, we performed rarefaction analyses that account for differences in sample size. In both subspecies, the number of detected alleles increased steadily with sample size and did not reach an asymptote within the sampled range (Fig. 1B).

### F1 sterility is polymorphic and asymmetric in wild populations

To assess variation in fertility among intersubspecific F1 hybrids, six males and 13 females from the Arzdorf population were mated with two inbred strains derived from wild mice sampled in Studenec: STUS (carrying the *Prdm9*^mus1^ allele and a high copy number of the *mir465* relative to the B6 reference-sized mir465 cluster) and STUF (carrying the *Prdm9*^mus5^ allele and a duplication of the mir465 cluster)(Jansa et al. 2025; Mukaj et al. 2020). These strains are known to produce sterile and fertile offspring, respectively, when crossed with the classical laboratory strain C57BL/10 J (B10), which represents a predominantly the *M. m. domesticus* genome (Yang et al. 2011) and carries the *Prdm9*^dom2^ allele (Kono et al. 2014). Notably, QTL analysis of a testcross [(STUS × STUF) × B10] revealed strong associations of male fitness components with the proximal end of chromosome 17 and the central region of chromosome X, consistent with the F1 sterility model (Vyskocilová et al. 2009).

**Fig. 2:**
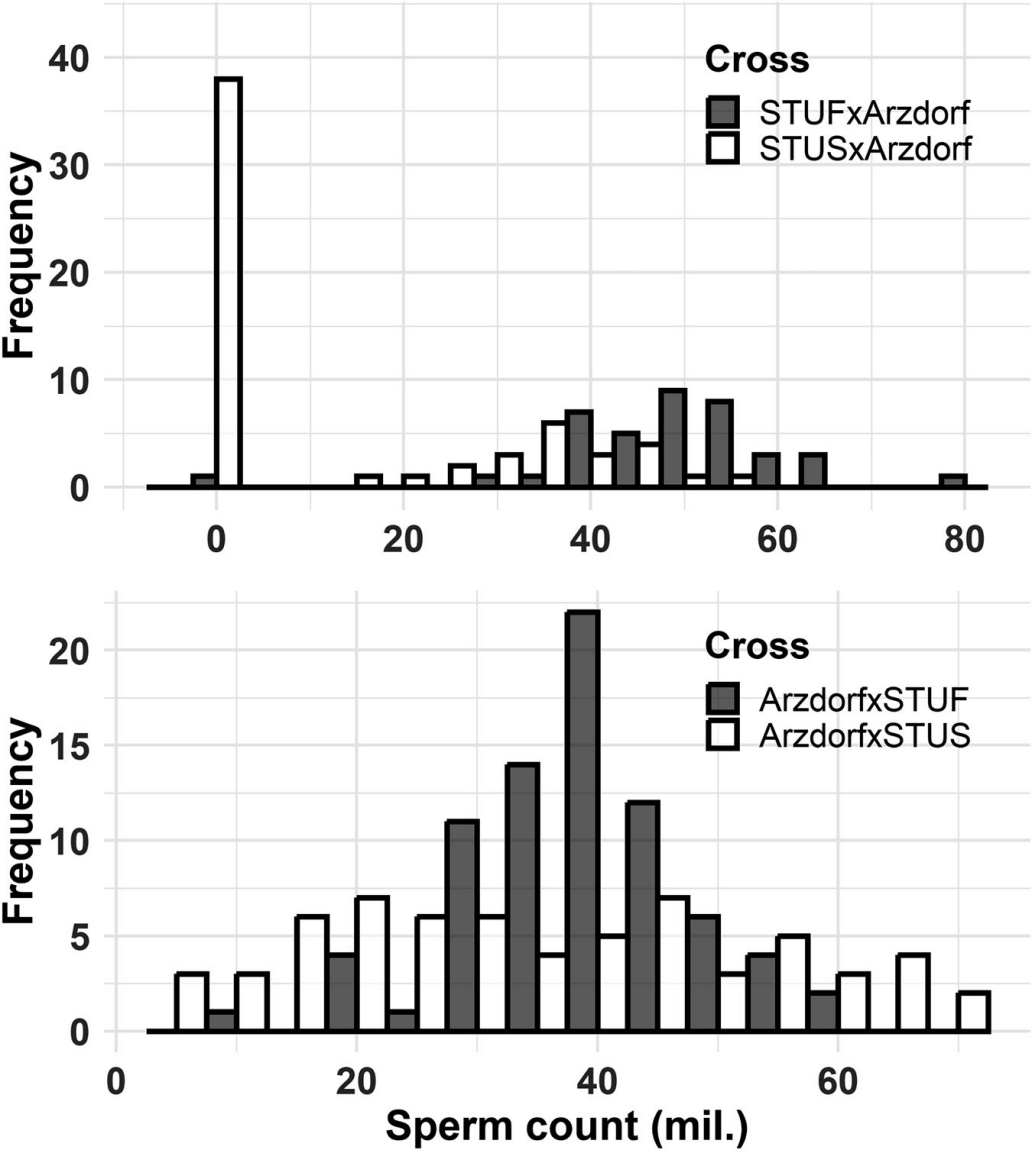
Distribution of sperm counts in F1 hybrids. The upper panel presents sperm counts in F1 hybrids from crosses between *M. m. musculus* strain females (STUF and STUS) and *M. m. domesticus* wild males (Arzdorf). The lower panel shows sperm counts from the reciprocal cross, involving *M. m. domesticus* wild females and *M. m. musculus* strain males.

The sperm count in 240 F1 hybrids varied greatly in both cross directions, ranging from complete azoospermia to 81.5 million sperm in the epididymis (Fig. 2; Table S1). Crosses with STUF mice produced fertile males, irrespective of the cross direction, with only one exception. Sterility in STUS mice was asymmetric between crosses, occurring almost exclusively in STUS females × Arzdorf males (38 sterile vs. 22 fertile), while being negligible in the reciprocal cross (Arzdorf females × STUS males; 6 sterile vs. 58 fertile). A G-test confirmed the asymmetry, showing a highly significant difference (*P* < 0.001). Although the Arzdorf population is far from areas known to harbour chromosomal rearrangements (Piálek et al. 2005), we considered the possibility that such rearrangements could still affect F1 hybrid fertility. However, analysis of mitotic spreads revealed standard karyotypes and ruled out chromosomal rearrangements as the genetic basis of F1 sterility (Dataset S1).

As noted above, the wild derived *M. m. musculus* representative PWD strain defines *Prdm9*/*Mir465*-associated sterility. We therefore examined whether the phenotypes of sterile males in our crosses resemble those described in the F1 sterility model. To this end, three PWD females were crossed with an *M. m. domesticus* Arzdorf male that had previously produced both fertile and sterile F1 males when mated with two *M. m. musculus* STUS females (2 fertile and 7 sterile hybrids in total). The PWD × Arzdorf cross similarly segregated for fertility among F1 males (9 sterile and 5 fertile). Sterile males exhibited markedly reduced sperm counts in the epididymis and smaller testes, mirroring the phenotypes observed in the STUS × Arzdorf crosses (Fig. S1). However, since the Arzdorf male carried *Prdm9*^dom10/dom102^ alleles lacking the known sterility-inducing variants and was not included in subsequent QTL analyses, the phenotypic similarity alone cannot establish a shared genetic basis of sterility.

### QTLs of sterility map to X-linked loci and depend on backcross type

We investigated whether the genetic architecture of hybrid sterility is consistent with predictions of the *Prdm9*/*mir465* epistatic model. To map quantitative trait loci (QTL) underlying sterility, we selected two Arzdorf males known to produce sterile F1 offspring. Fertile (STUS × Arzdorf)F1 females were backcrossed to either Arzdorf or STUS males (carrying the “sterile” *Prdm9*^mus1^ allele). The first backcross (BC) type produced 64 males (SAA), while the second (SAS) generated 47 recombinant males. To assess fertility-related traits, we measured both testes and left epididymis weights, estimated sperm count, and recorded mean litter size for males mated at 60 and 75 days with C3Hb/Stus females. All traits exhibited vast variation in BC males: average testis size varied from 13.6 to 93.9 mg, epididymis weight from 9.5 to 33.0 mg, sperm count from 0.0 to 90.0 mil. and litter size from 0.0 to 12 newborns.

**Fig. 3:**
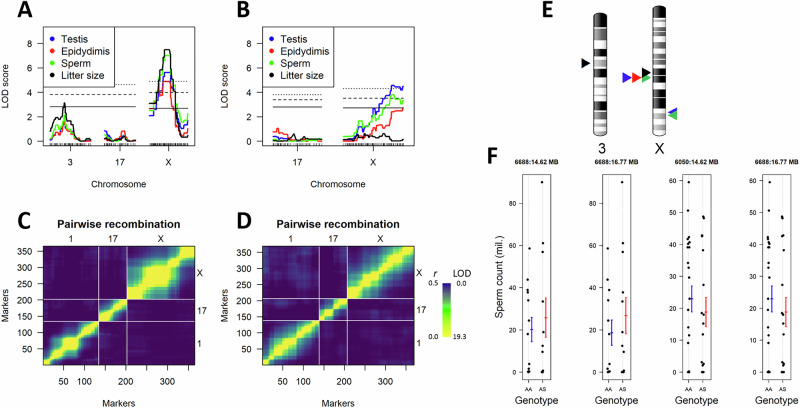
QTL mapping results. **A** LOD score for chromosomes associated with reproductive traits in the SAA backcross and **B** in the SAS backcross. Genome-wide significance thresholds from nonparametric statistics are shown as horizontal lines and were determined separately for autosomes and the X chromosome using 1000 permutations (solid line: *α* = 0.05; dashed line: α = 0.01; dotted line: *α* = 0.001). **C** Estimated recombination fraction for each pair of markers (*r*, shown above the diagonal) and LOD scores for the test of *r* = 0.5 (below the diagonal) for selected chromosomes in the SAA and (**D**) in the SAS backcross. The legend indicates ranges for recombination fractions *r* on the left and LOD scores on the right. **E** Schematic position of QTL on chromosomes 3 and X from nonparametric interval mapping, illustrating cross-dependent QTL signals (see Fig. S4 for details). QTL from the SAA and SAS crosses are shown on the left and right sides of each chromosome, respectively. While only the positions with maximal LOD scores are indicated, significant associations span broader regions of the X chromosome. QTL signal colours match those in (**A**, **B**). **F** Distribution of sperm counts against genotypes at two markers neighbouring the *Prdm9* gene, plotted separately for the male with dom3 allele (first two panels) and the male with different *Prdm9* alleles (the last two panels). In neither backcross was the phenotype associated with the markers (Kruskal-Wallis test, all *P* > 0.902). AA stands for Arzdorf (*M. m. domesticus*) alleles and AS for heterozygous *Prdm9*^Arzdorf/STUS^ allele. The chromosome ideograms in Fig. 2E were published by Wikimedia Commons (https://commons.wikimedia.org/w/index.php?title=File:Ideogram_house_mouse_chromosome.svg&oldid=915227600).

Most of the genome was not associated with variation in fertility-related traits (Fig. S2). Significant QTLs were detected on chromosome 3 and the X chromosome for SAA, and exclusively on the X chromosome for SAS (Fig. 3ABE, Table S2). The two backcross types differed in the positions of QTL on the X chromosome: in SAA, the QTL were located in the central region, whereas in SAS, they mapped to the distal part. Regardless of cross type, no QTL were mapped to chromosome 17, where the *Prdm9* gene is located. Along QTL, cross-type differences were further observed in recombination distribution, particularly along the X chromosome. SAA exhibited a nearly 40 Mb recombination cold spot in the central region of the X chromosome (GRCm39: 52.8–92.1 Mb) (Fig. 3CD, Fig. S3).

All crosses were conducted without prior knowledge of Prdm9 genotype. We therefore sequenced *Prdm9* in the parental males. Of the two males used in SAA backcrosses, one sired 24 males and carried the “sterile” *Prdm9*^dom3^ allele, whereas the other sired 40 males and was born in captivity to Arzdorf parents carrying *Prdm9*^dom102/dom104^ and *Prdm9*^dom10/dom102^ alleles (Dataset S1). We performed separate QTL mapping analyses for progeny derived from each male. In both subcrosses, significant LOD scores were detected exclusively on the central region of the chromosome X as in the SAA backcross (Fig. S4). Separate analysis of the “sterile” allelic combination *Prdm9*^dom3/mus1^ revealed no association with sperm count (Fig. 3F) or litter size (Kruskal-Wallis test, all *P* > 0.854). Similarly, no association was detected in the SAS cross for either sperm count or litter size (Kruskal–Wallis test, *P* = 0.691 and 0.284, respectively).

**Fig. 4:**
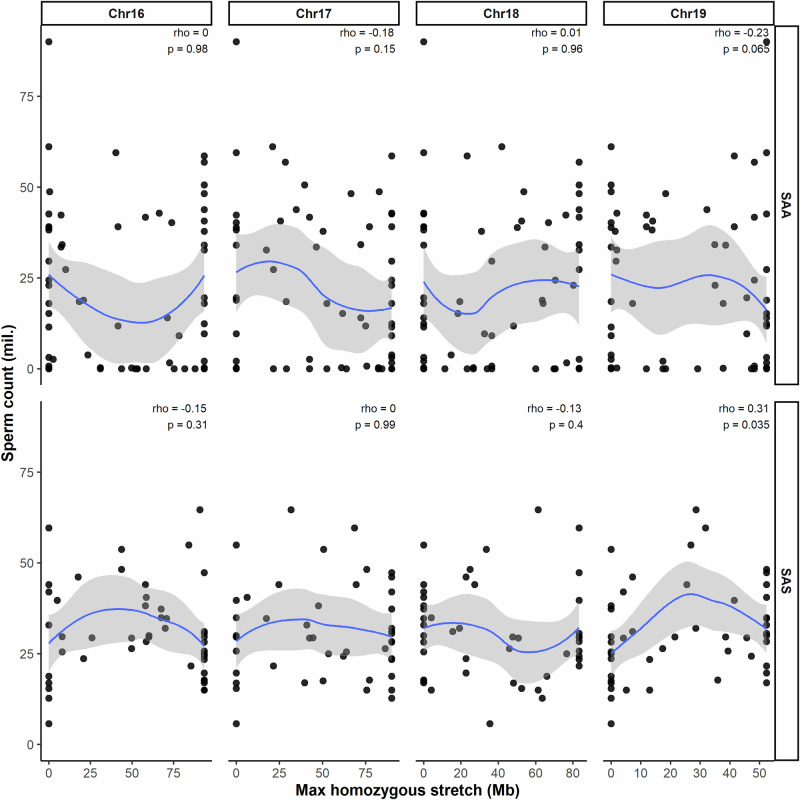
Relationship between homozygous block length on short autosomes and sperm count. Scatterplots showing sperm count plotted against the maximum length of homozygous genotype blocks (Mb) on chromosomes 16–19 in SAA and SAS backcross males. Each point represents an individual male. Spearman’s rank correlation coefficient (*rho*) is indicated in each panel.

Because extended autosomal homozygosity on short chromosomes has been shown to rescue synapsis and improve male fertility, we next tested whether the length of homozygous stretches on chromosomes 16–19 was associated with sperm count in our material. No significant association was detected in either backcross (Fig. 4). The only exception was a weak but significant positive correlation on chromosome 19 in the SAS cross (Spearman’s *ρ* = 0.31, *P* = 0.035; Fig. 4).

## Discussion

*Prdm9* has been widely recognized as a key factor influencing both recombination and hybrid male sterility in house mice (Forejt and Jansa 2023; Jansa et al. 2025; Mihola et al. 2009). At the same time, extensive work in *Mus musculus* has demonstrated that reproductive isolation is polygenic, with multiple autosomal and X-linked loci contributing to reduced fertility or sterility in a cross- and background-dependent manner. In well-characterized intersubspecific crosses, however, hybrid male sterility has consistently involved *Prdm9*-dependent mechanisms, even when additional loci or increased *musculus*/*domesticus* heterozygosity were shown to modulate the penetrance, severity, or developmental timing of spermatogenic failure (Flachs et al. 2012; Flachs et al. 2014; Forejt et al. 2021; Fotopulosova et al. 2024; Frayer and Payseur 2024; Reifová et al. 2023; Storchová et al. 2004; Wang et al. 2015).

To date, apparent departures from *Prdm9*-associated sterility have been explained by specific mechanistic factors, such as disruption of required *Prdm9*/*Hstx2* chromosome interactions or erosion of *Prdm9* binding asymmetry, rather than by the operation of a distinct, *Prdm9*-independent incompatibility pathway. In this context, our results extend current understanding by demonstrating that pronounced hybrid sterility can arise in intersubspecific crosses in which *Prdm9* does not contribute detectably to the phenotype. Instead, we find that reproductive isolation can be driven primarily by X-linked loci and, when considering litter size, by interactions between X-linked factors and an autosomal locus other than the *Prdm9* gene.

Two previously reported cases illustrate the mechanistic specificity of earlier exceptions to *Prdm9*-associated sterility. In the first, sterility QTL were examined in backcrosses between a female *M. m. domesticus* WSB (*Prdm9*^dom3^) and F1 hybrid males derived from PWD × CZECHII, both of which carry the *Prdm9*^mus1^ allele but segregate for fertility polymorphism within *M. m. domesticus* (Good et al. 2008b). Despite the presence of a sterility-associated ^Prdm9^ allele combination, no QTL was detected on chromosome 17. This absence can be explained by disruption of the *Prdm9*/*Hstx* chromosome interaction, as all backcross progeny inherited the X chromosome from the *domesticus* mother, whereas sterility in the original F1 model requires interaction with the *musculus*-derived *Hstx2* locus. Notably, this study adds another dimension to sterility polymorphism, demonstrating that when early-acting incompatibilities are disrupted, later-acting defects during spermatogenesis can become apparent, a phenomenon known in other cases (Coyne and Orr 2004; Vyskocilová et al. 2009).

A second exception has been reported in crosses between *M. m. musculus* strain PWK (*Prdm9*^mus1^) and *M. m. domesticus* strains SPLY and DBA/2J (*Prdm9*^dom3^ and *Prdm9*^dom2^, respectively) (Mukaj et al. 2020; Widmayer et al. 2020). In these cases, the expected *Prdm9* incompatibility did not induce sterility. This has been attributed to biased gene conversion during meiotic recombination, a process in which the repair of DNA double-strand breaks preferentially uses one parental DNA strand as a template. Over time, this non-Mendelian mechanism can erode *Prdm9* binding motifs on the other homolog, reducing the asymmetry of double-strand break formation and thereby mitigating the incompatibility (Baker et al. 2015; Lorenz and Mpaulo 2022; Smagulova et al. 2016). This mechanistic divergence contrasts with the incompatibilities observed in our study. Critically, the absence of significant QTL on chromosome 17 in our backcrosses—despite the presence of various *Prdm9* alleles—further supports the conclusion that the canonical *Prdm9*/*mir465* interaction is not the sole genetic determinant of hybrid sterility. Furthermore, in seven out of eight analyses, homozygosity on short autosomes showed no measurable association with male fertility. The only exception was a weak correlation on chromosome 19 in the SAS cross. However, even in this case, the loess curve does not increase gradually with the length of the homozygous stretch (Fig. 4), and can have, consequently, another causation.

Together, these findings argue against a universal, conserved genetic pathway underlying reproductive isolation in *Mus musculus*, and support the idea that hybrid sterility has evolved independently in different lineages. We refer to this alternative mode of hybrid sterility, in which incompatibility arises from X-linked loci with no detectable contribution of *Prdm9* under our experimental design, as *Prdm9*-independent X-linked sterility (PIXLS). Notably, the PIXLS model also contrasts with the recently described genetic architecture of interspecific hybrid sterility in cats, where eight autosomal and several X-linked candidate regions were detected (Davis et al. 2015). Although these cat lineages can still produce hybrids in captivity, their divergence time of ~7–10 My (unpublished data in Davis et al. 2015) suggests that some of the identified loci may reflect post-speciation incompatibilities rather than the primary causes of reproductive isolation.

Despite differences in genetic architecture between the PIXLS and *Prdm9*/*mir465* models, F1 males from both show similar phenotypes, including reduced testis size and azoospermia, as observed in crosses between STUS or PWD (mus1) and Arzdorf males. These phenotypes, along with the asymmetric sterility that is more pronounced in crosses involving female *musculus* × male *domesticus* than in the reciprocal direction, are consistent with previous studies (Davies et al. 2016; Forejt et al. 2021; Good et al. 2008b). Moreover, sterility segregates within both *M. m. domesticus* and *M. m. musculus* localities, a pattern also observed in the *Prdm9*/*mir465* model (Good et al. 2008b; Larson et al. 2018; Mukaj et al. 2020; Vyskocilová et al. 2009). Both models also conform to two rules of speciation. First, male sterility aligns with Haldane’s rule, which predicts that the heterogametic sex (males in mammals) is more often affected (Haldane 1922). Second, sterility QTL in the PIXLS model map to the X chromosome, supporting the large X-effect observations that X-linked genes disproportionately contribute to hybrid sterility or inviability in male-heterogametic species (Coyne and Orr 1989; Coyne and Orr 2004). The phenotypic similarity between models with distinct genetic bases underscores the need for deeper genetic dissection since phenotype alone cannot reveal the underlying causes of hybrid incompatibility.

Earlier studies dissecting the genetic basis of sterility using backcross populations typically relied on single-direction crosses involving mus1 and dom2/dom3 alleles (Dzur-Gejdošová et al. 2012; Storchová et al. 2004). While different studies reported numerous autosomal QTLs for different sterility phenotypes, they consistently detected major QTL in regions spanning the *Prdm9* and *Hstx*/*mir465* loci. An exception was an F2 cross between *M. m. castaneus* and *M. m. domesticus*, which revealed a distinct architecture involving autosomal, X-linked, and pseudoautosomal loci, with a strong dependence on cross direction (White et al. 2011; White et al. 2012). In our study, sterility-associated QTLs mapped almost exclusively to the X chromosome, with their position shifting depending on backcross direction: central in SAA and distal in SAS. In both cases, QTL extended across most of the X chromosome, which may partly reflect the use of nonparametric test with moderate numbers of BC males. However, at least some of this variation likely reflects natural mechanisms of sterility as suggested in a study with reciprocal introgression of the X chromosome between wild *musculus* and *domesticus*. In experiments with consomic strains carrying proximal, central and distal regions of the X chromosome, introgression of the *musculus* X chromosome into a *domesticus* genetic background, but not the reverse, produced male-limited sterility. Relative testis weight and sperm count were negatively correlated with the length of the introgressed *musculus* X, with strongest detrimental effects observed when all three regions were translocated (Good et al. 2008a). Consistently with our study, QTL of testis weight mapped to different regions depending on cross direction. This direction-dependent mapping, now observed across three subspecies, points to a broader principle in hybrid male sterility and highlights the importance of reciprocal cross designs. Moreover, the recombination cold spot on the X chromosome in the SAA backcross suggests the involvement of structural or epigenetic factors that merit further investigation. The complexity of genetic basis of sterility can increase when two mouse strains with *Prdm9*^mus1/dom3^/*Hstx*-associated sterility model were analysed for genome-wide misexpression in testis from F1 and F2 hybrids (Turner et al. 2014). Many eQTL cluster into eleven ‘hotspots,’ seven of which co-localize with several autosomal QTL for sterility phenotypes identified in the cross (Turner et al. 2014; White et al. 2011).

Population genetic data reveal extensive *Prdm9* allelic variation in both *M. m. domesticus* and *M. m. musculus* populations. This high diversity is consistent with previous population-level studies, which also reported broad allelic variability in *M. m. domesticus* (Marín-García et al. 2024, SI Appendix, Dataset S2), suggesting limited or no positive selection at local scales. While some populations in that study were fixed for a single allele, this is likely the result of founder effects, a common outcome in house mouse colonization dynamics. Overall, the observed allelic diversity challenges the view that *Prdm9*-dependent Dobzhansky–Muller incompatibilities are driven by strong positive selection. If selection were promoting the fixation of incompatible alleles to reinforce nascent species barriers, we would expect reduced allelic variation. Instead, our findings support an alternative intra-genomic Red Queen model, driven by the interplay between two antagonistic forces: biased gene conversion leading to hotspot extinction (the hotspot conversion paradox), followed by selection favouring novel *Prdm9* alleles recognizing new sequence binding motifs (Genestier et al. 2024). This rapid mutational turnover can eventually generate incompatible alleles that can interact with other regulating factors affecting spermatogenesis; however, due to erosion of PRDM9 binding sites, such incompatibilities might be evolutionary transient. Importantly, with this study, only 27 alleles out of 166 (Dataset S2) have been tested in fertility assays.

The rarefaction analysis indicates that current sampling does not fully capture *Prdm9* allelic diversity at either the local or subspecies level. While common alleles appear to be well represented, the continued increase of the curves suggests that many low-frequency alleles remain unsampled. As a result, observed diversity estimates should be regarded as conservative, and we refrain from quantitative extrapolation of total *Prdm9* allelic richness across subspecies.

Interpreting results within the context of the house mouse hybrid zone (HMHZ) is less straightforward, as the two sterility models imply different underlying genetic architectures. Genetic analyses across multiple zone replicates consistently show that (i) X-linked markers experience stronger selection compared with autosomal loci (Janoušek et al. 2012; Macholán et al. 2007; Tucker et al. 1992), (ii) gene flow along the X chromosome is heterogeneous, being most restricted in its central region (Dufková et al. 2011; Macholán et al. 2011; Payseur et al. 2004), and (iii) the *musculus* Y chromosome introgresses into the *domesticus* genetic background, likely through interactions with X-linked loci (Baird et al. 2023; Macholán et al. 2008; Macholán et al. 2019). As noted above, a significant reduction in sperm quality has been reported in hybrid zone populations, but F1 mice and fully sterile males are largely absent in the centre of the HMHZ (Albrechtová et al. 2012; Turner et al. 2012).

Two of the three prerequisites defining the *Prdm9*/*mir465* model appear to be met in the HMHZ. Although F1 hybrids are largely absent in the centre of the zone, allelic variants of *Prdm9* within each subspecies remain compatible, enabling sterility-associated alleles to spread within their native genomic backgrounds and meet at the contact edges. Given that the dom3 and mus1 alleles are among the most common *Prdm9* variants in wild mice (Dataset S2), their occurrence in the HMHZ is plausible. However, the long-term persistence of incompatible alleles is uncertain. Datasets S1 and S2 reveal high allelic variation within local populations, which is expected to be even greater in the HMHZ. Because males carrying sterility-inducing *Prdm9* alleles experience reduced fitness, and because numerous alternative, fertility-maintaining alleles are available, selection should favour the rapid purging of incompatible variants and their replacement by less deleterious ones. At present, no high-resolution data are available on *Prdm9* variation within the HMHZ, limiting our ability to infer its evolutionary dynamics in situ. Moreover, after more than two millennia of continuous gene flow and recombination, introgressed parental haplotypes are expected to have been broken into short ancestry tracts. This likely undermines the third prerequisite of the *Prdm9*/*mir465* model, namely, the presence of long heterozygous genomic segments on short chromosomes that is considered necessary for hybrid sterility to arise (Gregorová et al. 2018). Finally, the interacting *mir465* cluster is located on the X chromosome, which experiences stronger selection and more restricted introgression than the autosomes.

In contrast, the PIXLS model involves two or more loci on the X chromosome, consistent with genetic footprints of selection observed in the HMHZ. Beyond this, further discussion remains speculative until the loci underlying the PIXLS model are identified. Notably, the coexistence of a second sterility model may complicate the mapping of sterility QTL in the HMHZ, as both models can affect fitness independently or interactively within the hybrid zone. Broader functional validation of candidate allelic variants in the laboratory, together with detailed analysis of their distribution across the hybrid zone, will be essential to evaluate the general role of ‘speciation genes’ in reproductive isolation.

## Materials and methods

**Mice.** Wild *Mus musculus domesticus* and *M. m. musculus* were sampled in Arzdorf [50° 37’ N, 7° 05’ E], Germany, and Studenec [49° 12’ N, 16° 04’ E], Czech Republic, respectively. Wild-derived *M. m. musculus* strains STUF and STUS originate from Studenec and are maintained at the breeding facility of the Institute of Vertebrate Biology in Studenec (Piálek et al. 2008; Piálek et al. 2025). The strains used in experiments were at least in the 8th generation of brother–sister mating. All mice were housed under standard conditions with a 14/10 h light/dark cycle and free access to food and water. The breeding facility is licensed for housing house mice (permit no. MZE-50144/2022-13143).

**Experimental mating.** To analyse sterility-associated polymorphism in a wild population of *M. m. domesticus* (Arzdorf), six males and thirteen females were mated with males and females from the wild-derived *M. m. musculus* STUF and STUS strains. Four groups of F1 hybrid males were generated: Arzdorf × STUF (*n* = 77), Arzdorf × STUS (*n* = 64), STUF × Arzdorf (*n* = 39), and STUS × Arzdorf (*n* = 99) (Table S1). Note that in all cross abbreviations, the female parent is listed first.

The genetic basis of incompatibilities was analysed using backcrossed males. F1 females derived from the cross producing sterile F1 males (STUS × Arzdorf) were backcrossed to either *M. m. domesticus* (backcross SAA) or STUS males (*M. m. musculus*; backcross SAS). These crosses yielded 64 BC males in the former and 47 BC males in the latter group.

All males were weaned individually at 20 days of age. At 60 and 75 days, they were each mated to C3Hb females for 15 days, and fertility was estimated as the mean value from the two mating trials. Males were isolated at 90 days of age and dissected at 100 days.

**Spermatogenesis.** Immediately after cervical dislocation of hybrid males, morphometric data were collected, and both testes and the left epididymis were dissected and weighed. Sperm was counted from the entire left epididymis using a Bürker hemocytometer, following the protocol described in Vyskocilová et al. (2009). Spleen was preserved in 96% ethanol.

**Karyotype analysis.** Chromosome numbermsa-stained mitotic spreads prepared from bone marrow cells after 20 min of in vivo colchicine treatment (Gosden 1994).

***Prdm9*** **sequencing.** We performed Sanger sequencing of the zinc finger (ZnF) domain of the *Prdm9* gene, following the protocol published by Piálek et al. (2025). Allelic classification and nomenclature, based on the most amino-acid polymorphic sites across all ZnFs and presented in Datasets S1, S2, follow Mukaj et al. (2020). Four sequences from *M. m. musculus* were retrieved from GenBank (Larson et al. 2022; Mukaj et al. 2020; Piálek et al. 2025).

**Rarefaction analysis.** We assessed sampling completeness and the accumulation of *Prdm9* allelic diversity using rarefaction analysis. Presence–absence data were compiled separately for *M. m. domesticus* and *M. m. musculus* from published and newly identified alleles (Dataset S2). Individuals were randomly subsampled without replacement across increasing sample sizes, and the expected number of distinct *Prdm9* alleles was estimated using 1000 bootstrap resamplings to generate mean rarefaction curves and confidence intervals. Curves were compared at equal sampling depths to account for differences in sample size between subspecies. As the curves did not approach saturation, no asymptotic extrapolation of total allelic richness was performed.

**Molecular screen of backcross progeny.** To design a panel of genetic markers for QTL mapping, we first genotyped 143,259 SNPs in one male and one female from each of 24 wild-derived strains using the Giga Mouse Universal Genotyping Array (GigaMUGA, Morgan et al. 2015). We then selected 5000 SNPs, distributed approximately evenly across the genome, and used the SeqSNP service (LGC, Queens Road, Middlesex, UK) to genotype six parental individuals, two F1 hybrids, and 111 backcross animals. We manually removed invariable and non-informative markers from the resulting genotype array. For QTL analysis, we ultimately used 1837 SNPs in the SAA cross and 1914 SNPs in the SAS cross.

**Statistical analysis.** All analyses were performed in the RStudio, version 2023.06.0 (Posit 2019). We first used sperm data to delimit F1 males into two groups linked to fertility. The valley-based threshold splitting method was used for separating the bimodal distribution of sperm counts into “sterile” and “fertile” males. The ‘density’ function from ‘stats’ R library was used for Kernel density estimation and the function ‘which.min’ was invoked to find local minimum between two peaks (Fig. S5). Shapiro-Wilk test detected that sperm count is not normally distributed; consequently, a Wilcoxon nonparametric test was used to analyse differences in median sperm counts between groups of F1 males. LOD scores of sterility-associated QTL in backcross progeny were estimated by the nonparametric interval function ‘scanone, (model = ”np”)’ implemented in R/qtl (Broman and Sen 2009).

**Analysis of homozygosity and sperm count.** For each backcross individual, the maximum length of contiguous homozygous genotype blocks was calculated separately for chromosomes 16–19 based on the physical position of SNP markers. Marker genotypes were ordered by chromosome and physical position, and the longest uninterrupted stretch of homozygous markers per chromosome was identified. Block length was defined as the physical distance (Mb) between the first and last SNP within each contiguous homozygous segment.

To test whether homozygosity was associated with male fertility, we analysed the relationship between maximum homozygous block length and sperm count separately for the SAA and SAS backcrosses. For each chromosome, Spearman’s rank correlation coefficients (*ρ*) were calculated between sperm count and block length. *P*-values were obtained using ‘cor.test’ function in R, with exact tests disabled due to ties in the data.

## Supplementary information

Supplementary material

Dataset 1

Dataset 2

## Data Availability

**Data accessibility.** Supplementary datasets, tables and figures are available at Heredity online. *Prdm9* sequences are deposited in GenBank (accession numbers PV833228–PV833253).
