# Supplementary material for: Beyond the Prdm9 model: independent evolution of hybrid male sterility in house mice
Source: Heredity (Edinb). 2026 Mar 12;135(5):323–31. doi: 10.1038/s41437-026-00834-9 (PMC13219761; doi:10.1038/s41437-026-00834-9)
Supplement: Supplementary file 1 — Supplementary material [file 41437_2026_834_MOESM1_ESM.docx]

**Supporting information for**

Beyond the *Prdm9* model: Independent evolution of hybrid male sterility in house mice

Pavla Klusáčková^a^, Agata Woźniewska^b^, Petra Dufková^a^, Beth L. Dumont^c^, Jan M. Wójcik^b^, Jaroslav Piálek^a,*^

^*^To whom correspondence should be addressed: E-mail: [jpialek@ivb.cz](mailto:jpialek@brno.cas.cz)

**This PDF file includes:**

Figures S1 to S5

Tables S1 to S2

**Other supporting materials for this manuscript include the following:**

Dataset S1 and S2 in Excel files

Fig. S1.


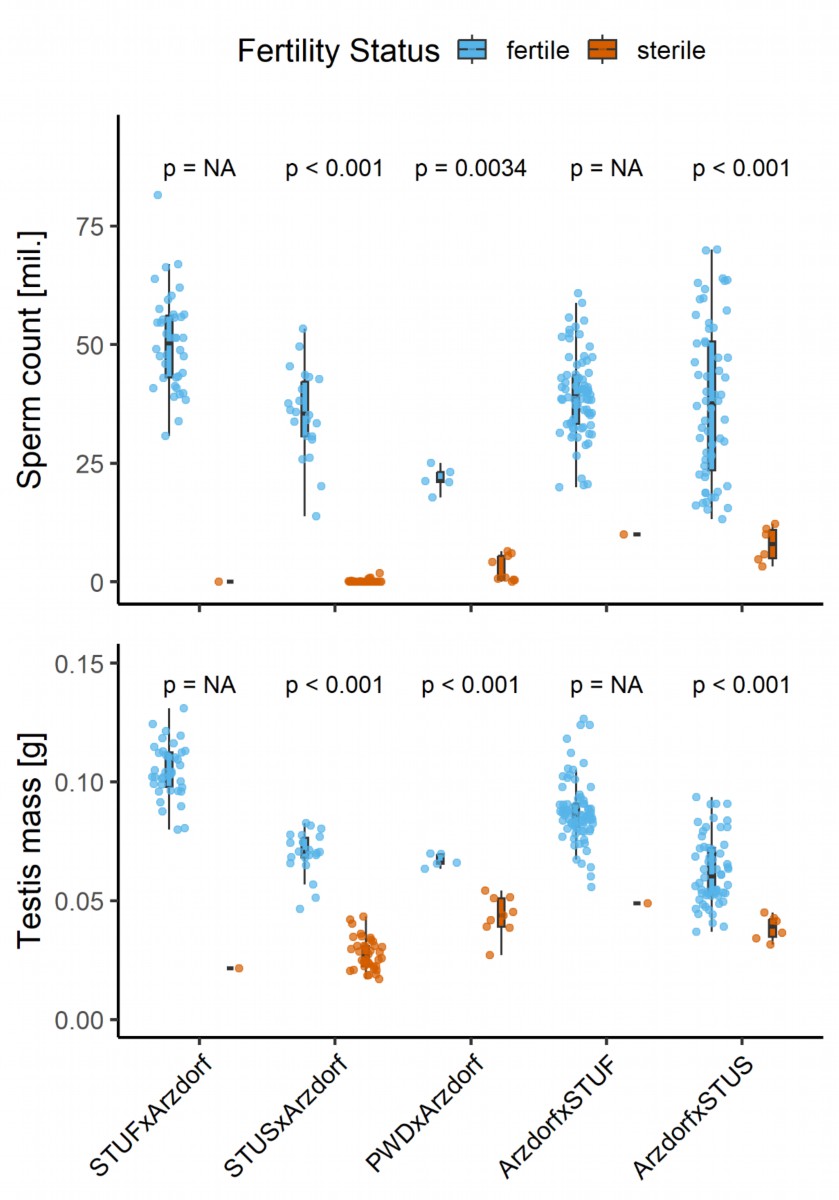


Fig. S1. **Fertility data for sperm count (upper panel) and average testis size (lower panel)** in F1 hybrids. Each dot represents an individual measurement. Male fertility status, indicated by colour, is based on threshold values derived from sperm count. Differences between sterile and fertile males within each cross were tested using the Wilcoxon nonparametric test. The threshold value to split fertility of males into two categories was estimated using valley-based threshold splitting method and is illustrated in Fig. S5.

Fig. S2.


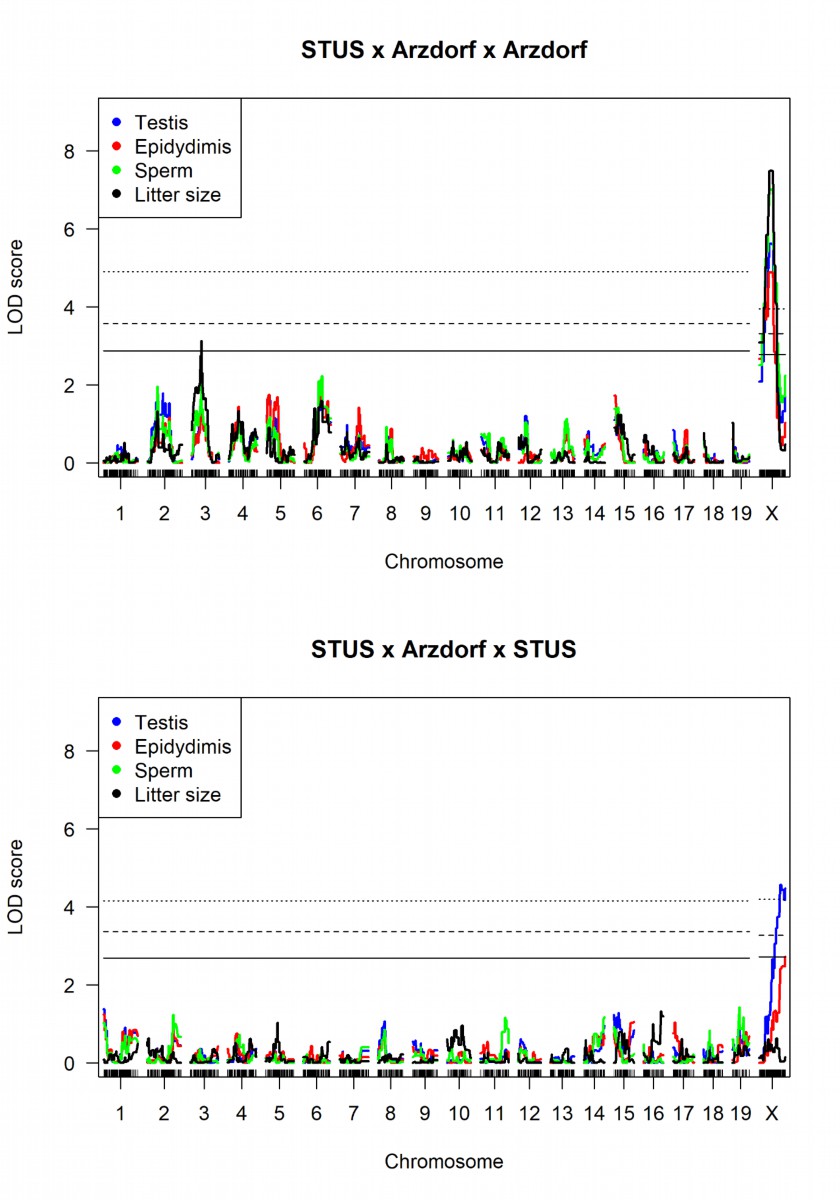


Fig. S2. QTL mapping of reproductive traits in all chromosomes for males from both reciprocal backcrosses. Nonparametric interval mapping was used to estimate LOD scores. Genome-wide significance thresholds based on nonparametric statistics are shown as horizontal lines and were calculated separately for autosomes and the X chromosome using 1,000 permutations (solid line: α = 0.05; dashed line: α = 0.01; dotted line: α = 0.001)..

Fig. S3.


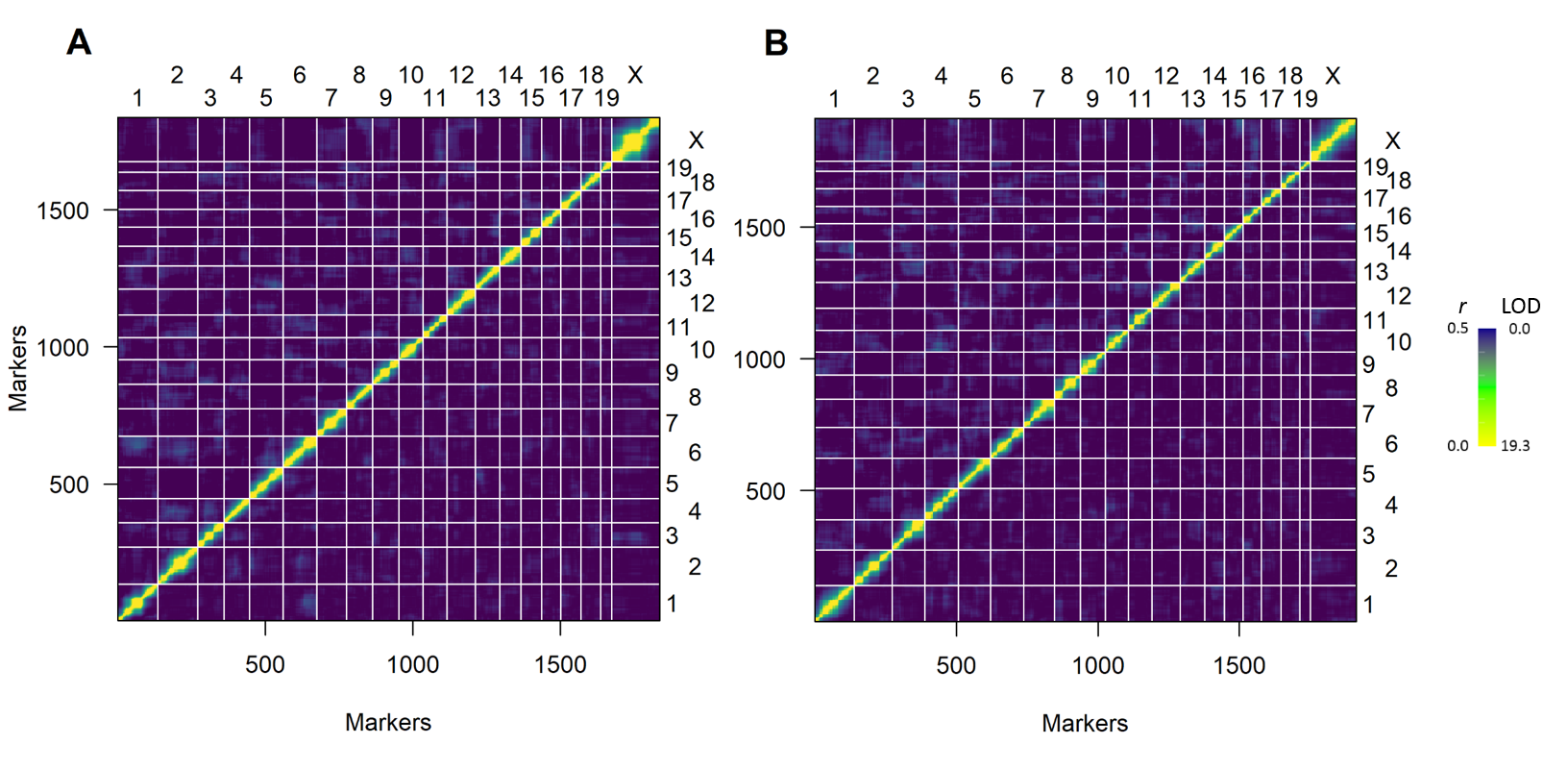


Fig. S3. Distribution of recombination fractions across all chromosomes (upper left) and LOD scores for independent recombination (lower right) for all pairs of markers in (A) SAA: ♀(STUS × Arzdorf) × ♂Arzdorf and (B) SAS: ♀(STUS × Arzdorf) × ♂STUS. A pronounced recombination cold spot in the central region of the X chromosome in the SAA cross may hinder QTL mapping in this region.

Fig. S4.


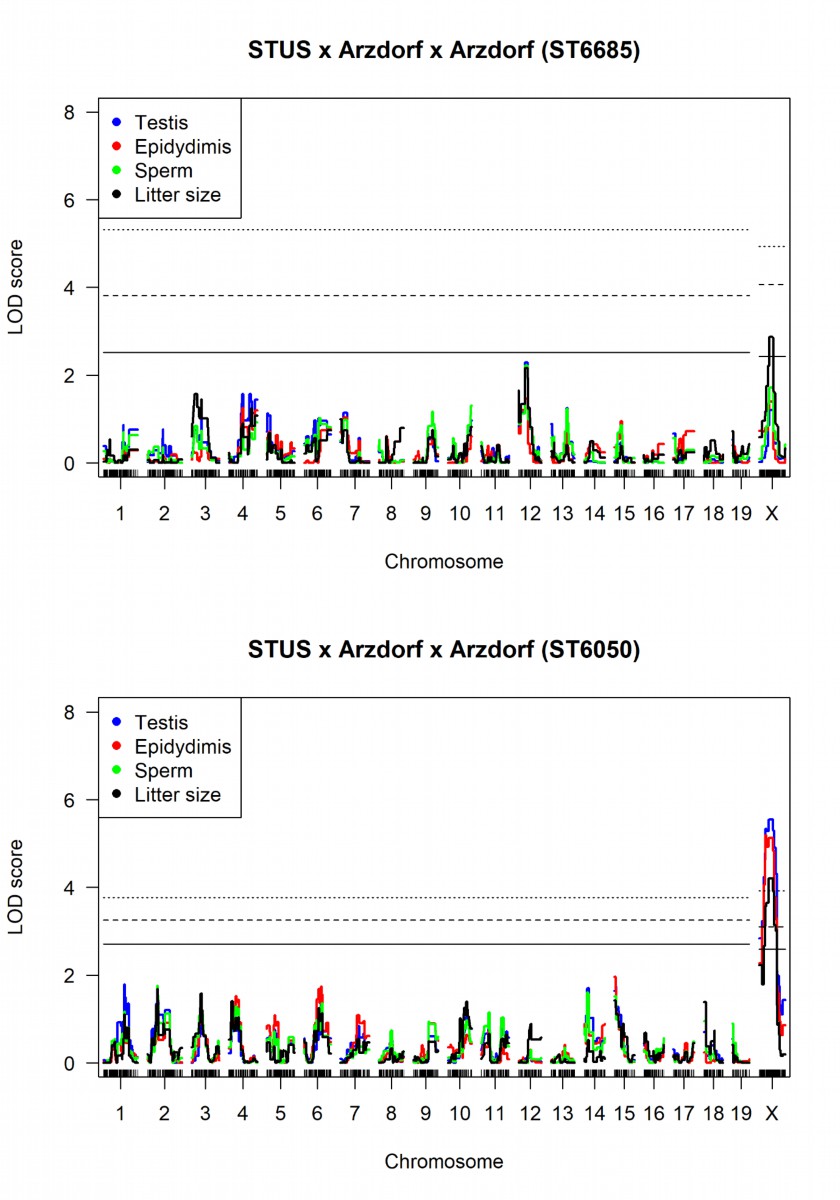


Fig. S4. LOD scores for QTLs associated with reproductive traits in the SAA backcross, plotted separately for the two male parents used in the experiments. Nonparametric interval mapping was used to estimate LOD scores. Genome-wide significance thresholds based on nonparametric statistics are shown as horizontal lines and were calculated separately for autosomes and the X chromosome using 1,000 permutations (solid line: α = 0.05; dashed line: α = 0.01; dotted line: α = 0.001).

Fig. S5.


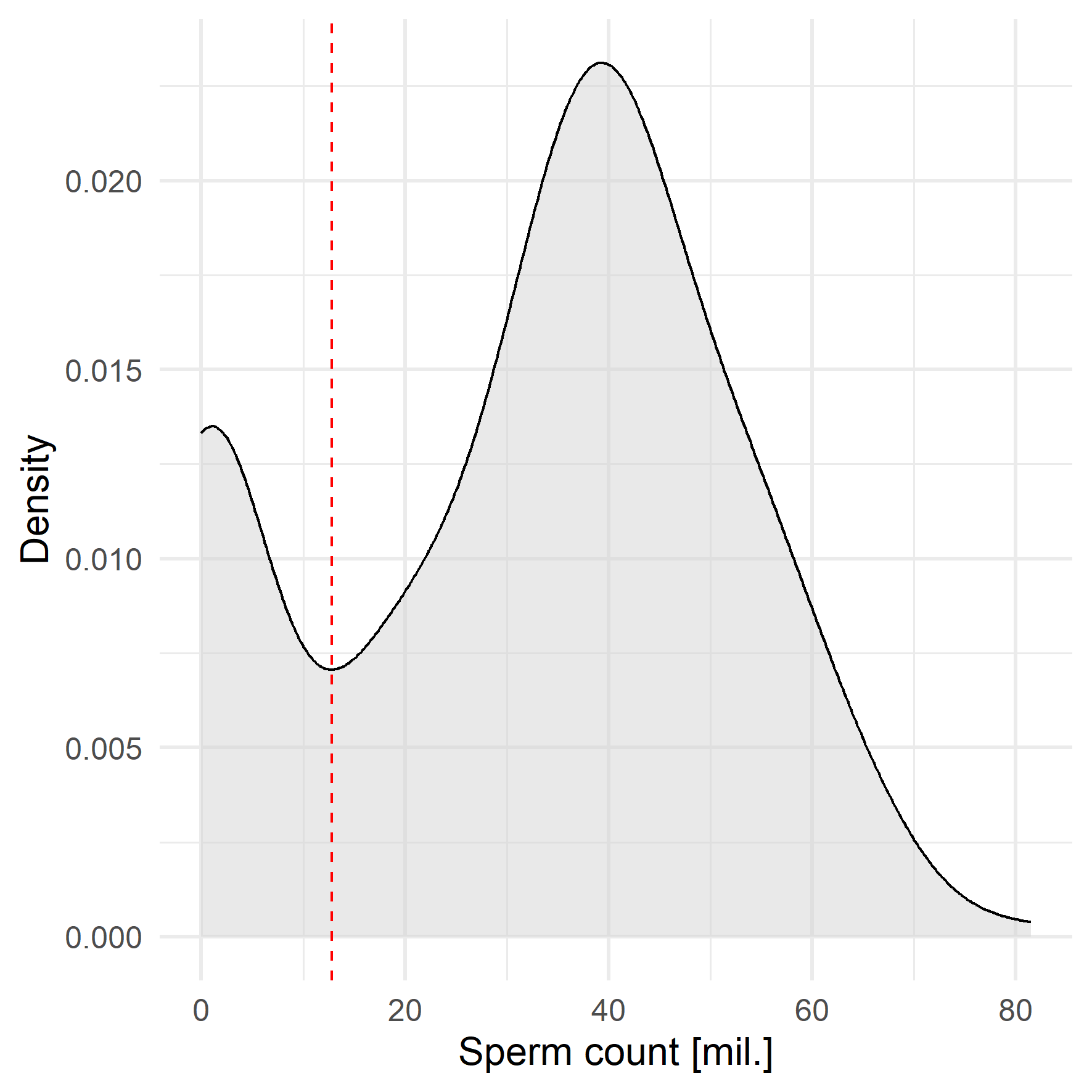


Fig. S5. Valley-based threshold detects the value 12.78 mil splitting “sterility” and “fertility” in bimodal sperm distribution in F1 hybrids.

Table S1. Numbers of sterile (0–12.78 mil.) and fertile (> 12.78 mil.) F1 hybrids from Studenec females × Arzdorf males (upper part of table), and Arzdorf females × Studenec males (lower part of table). The sperm count threshold used to define sterility and fertility is detailed in Materials and Methods and illustrated in Fig. S5. STUF and STUS are representatives of *M. m. musculus*, Arzford originate from *M. m. domesticus* population.

| Arzdorf | STUF | |  | STUS | | N mice |
| --- | --- | --- | --- | --- | --- | --- |
| Males | **0–12.8** | **>12.8** |  | **0–12.8** | **>12.8** |  |
| 5065 | 0 | 8 |  | 1 | 9 |  |
| 5066 | 0 | 8 |  | 0 | 10 |  |
| 5067 | 1 | 5 |  | 7 | 2 |  |
| 6685 | 0 | 10 |  | 20 | 0 |  |
| 6686 | 0 | 3 |  | 1 | 1 |  |
| 6687 | 0 | 4 |  | 9 | 0 |  |
| Males | **1** | **38** |  | **38** | **22** | 99 |
|  |  |  |  |  |  |  |
| Females | **0–12.8** | **>12.8** |  | **0–12.8** | **>12.8** |  |
| 5068 | 0 | 14 |  | 0 | 8 |  |
| 5069 | 1 | 5 |  | 4 | 7 |  |
| 5070 | 0 | 3 |  |  |  |  |
| 5071 | 0 | 3 |  | 0 | 2 |  |
| 5072 | 0 | 9 |  | 1 | 5 |  |
| 5073 | 0 | 9 |  |  |  |  |
| 5074 | 0 | 6 |  |  |  |  |
| 6688 | 0 | 8 |  | 1 | 6 |  |
| 6690 |  |  |  | 0 | 7 |  |
| 6692 | 0 | 7 |  | 0 | 12 |  |
| 6693 |  |  |  | 0 | 3 |  |
| 6694 | 0 | 5 |  |  |  |  |
| 6695 | 0 | 7 |  | 0 | 8 |  |
| Females | **1** | **76** |  | **6** | **58** | 141 |
|  |  |  |  |  |  |  |
| N mice |  | 116 |  |  | 124 | 240 |

Table S2. QTL interval mapping for reproductive traits with nonparametric distributions. Locus names correspond to either marker included in the GigaMUGA SNP array or pseudo-markers generated by the R/qtl software. The **SAA** cross refers to a (STUS × Arzdorf) × Arzdorf backcross, while **SAS** refers to a (STUS × Arzdorf) × STUS backcross.

| Chromosome | position (cM) | position (Mb) | LOD | Probability | Locus | Cross | Trait |
| --- | --- | --- | --- | --- | --- | --- | --- |
| X | 38.174 | 94.205 | 5.646 | 0.000 | JAX00182107 | SAA | Testis |
| X | 38.174 | 94.205 | 4.890 | 0.000 | JAX00182107 | SAA | Epididymis |
| X | 38.174 | 94.205 | 7.018 | 0.000 | JAX00182107 | SAA | Sperm |
| 3 | 27.110 | 63.142 | 3.127 | 0.023 | UNCHS008870 | SAA | Litter size |
| X | 36.840 | 88.940 | 7.484 | 0.000 | JAX00715308 | SAA | Litter size |
| X | 61.075 | 139.039 | 4.609 | 0.000 | cX.loc60 | SAS | Testis |
| X | 57.779 | 140.119 | 3.815 | 0.004 | UNC31386645 | SAS | Sperm |
